# *De Novo* Assembly of a Field Isolate Genome Reveals Novel *Plasmodium vivax* Erythrocyte Invasion Genes

**DOI:** 10.1371/journal.pntd.0002569

**Published:** 2013-12-05

**Authors:** James Hester, Ernest R. Chan, Didier Menard, Odile Mercereau-Puijalon, John Barnwell, Peter A. Zimmerman, David Serre

**Affiliations:** 1 Genomic Medicine Institute, Cleveland Clinic Lerner Research Institute, Cleveland, Ohio, United States of America; 2 Unité d'Epidémiologie Moléculaire, Institut Pasteur du Cambodge, Phnom Penh, Cambodia; 3 Unité d'lmmunologie Moléculaire des Parasites, Institut Pasteur, Paris, France; 4 Division of Parasitic Diseases and Malaria, Centers for Disease Control and Prevention, Atlanta, Georgia, United States of America; 5 Center for Global Health and Diseases, Case Western Reserve University, Cleveland, Ohio, United States of America; Barcelona Centre for International Health Research (CRESIB) and Institució Catalana de Recerca i Estudis Avançats (ICREA), Spain

## Abstract

Recent sequencing of *Plasmodium vivax* field isolates and monkey-adapted strains enabled characterization of SNPs throughout the genome. These analyses relied on mapping short reads onto the *P. vivax* reference genome that was generated using DNA from the monkey-adapted strain Salvador I. Any genomic locus deleted in this strain would be lacking in the reference genome sequence and missed in previous analyses. Here, we report *de novo* assembly of a *P. vivax* field isolate genome. Out of 2,857 assembled contigs, we identify 362 contigs, each containing more than 5 kb of contiguous DNA sequences absent from the reference genome sequence. These novel *P. vivax* DNA sequences account for 3.8 million nucleotides and contain 792 predicted genes. Most of these contigs contain members of multigene families and likely originate from telomeric regions. Interestingly, we identify two contigs containing predicted protein coding genes similar to known *Plasmodium* red blood cell invasion proteins. One gene encodes the reticulocyte-binding protein gene orthologous to *P. cynomolgi* RBP2e and *P. knowlesi* NBPXb. The second gene harbors all the hallmarks of a *Plasmodium* erythrocyte-binding protein, including conserved Duffy-binding like and C-terminus cysteine-rich domains. Phylogenetic analysis shows that this novel gene clusters separately from all known *Plasmodium* Duffy-binding protein genes. Additional analyses showing that this gene is present in most *P. vivax* genomes and transcribed in blood-stage parasites suggest that *P. vivax* red blood cell invasion mechanisms may be more complex than currently understood. The strategy employed here complements previous genomic analyses and takes full advantage of next-generation sequencing data to provide a comprehensive characterization of genetic variations in this important malaria parasite. Further analyses of the novel protein coding genes discovered through *de novo* assembly have the potential to identify genes that influence key aspects of *P. vivax* biology, including alternative mechanisms of human erythrocyte invasion.

## Author Summary

*Plasmodium vivax* is responsible for most malaria cases outside Africa, but is poorly understood, as the parasite is difficult to study *in vitro*. Genome sequencing studies offer a novel and exciting opportunity to better understand this parasite but, so far, have directly mapped reads onto the reference genome sequence generated from a single *P. vivax* strain. Here, we use sequence data generated from a field isolate to reconstruct long DNA sequences without relying on the reference genome. Our analyses reveal many *P. vivax* DNA sequences that are absent from the reference genome and contain 792 predicted genes. One of these novel genes encodes a predicted protein similar to known *Plasmodium* proteins involved in red blood cell invasion. This new gene is present in all *P. vivax* strains sequenced so far, except for the strain used to generate the reference genome, and is transcribed in blood-stage parasites. Overall, our analyses show that the catalogue of *P. vivax* genes was incomplete and that potentially important genes have been missed. We notably identified one putative invasion gene that seems functional and could dramatically change our understanding of the mechanisms determining red blood cell invasion by this important malaria parasite.

## Introduction

Despite being responsible for several million cases of clinical malaria every year, we still know very little about the biology of *Plasmodium vivax*. An important limitation of *P. vivax* research is the lack of continuous *in vitro* propagation that hampers development of functional assays and collection of sufficient amounts of biological material for studying this parasite. To circumvent these constraints, researchers often rely on materials derived from *P. vivax* strains that have been adapted to non-human primates (typically *Saimiri* and *Aotus* monkeys). These monkey-adapted strains are an essential resource for the *P. vivax* research community as they can provide large amount of parasites for protein and nucleic acid studies and *ex vivo* assays. It is therefore not surprising that monkey-adapted strains have played a prominent role in the identification of *P. vivax* proteins responsible for red blood cell (RBC) invasion [1]–[3]. While analyses of monkey-adapted strains have yielded invaluable insights, only a small number of strains are available [4] which limits their use for studying *P. vivax* biological diversity. In addition, development of monkey-adapted strains requires the parasite to switch hosts and adapt to a novel *in vivo* environment, including differences in immune systems and RBC surface proteins. It is therefore not clear whether monkey-adapted strains provide an unbiased perspective on the biology of *P. vivax in vivo*.

Genomic studies leveraging developments in sequencing technology have the potential to complement *in vitro* studies and to fill some of the gaps in our understanding of *P. vivax* biology. Recent whole genome sequencing studies of monkey-adapted strains [5] and field isolates [6], [7] have enabled genome-wide characterization of single nucleotide polymorphisms (SNPs). However, these studies have so far relied on mapping short reads generated by massively parallel sequencing onto the *P. vivax* reference genome sequence that was generated using DNA from a single strain, the monkey-adapted Salvador I strain [8]. These previous studies were therefore only able to analyze variations at genomic loci present in the Salvador I strain and would have overlooked any loci deleted in this genome. We have shown that polymorphic DNA sequence rearrangements exist among *P. vivax* strains and include large deletions containing annotated protein coding genes [9]. Thus, it is possible that the Salvador I strain lacks genomic loci present in other *P. vivax* parasites. In this regard, it is interesting to note that recent genome sequencing of the closely related *P. cynomolgi* parasite highlighted several invasion protein genes without known orthologous genes in the *P. vivax* reference genome [10].

Whole genome sequencing data also provide an opportunity to circumvent these shortcomings. Instead of directly mapping massively parallel sequencing reads onto a reference genome sequence, one can *de novo* assemble them into long contiguous DNA sequences or “contigs” [11]. These contigs can then be compared to the reference genome sequence to identify sequence rearrangements and novel DNA sequences. Here, we apply this approach to *P. vivax* and report the *de novo* genome assembly of a *P. vivax* field isolate from Cambodia. Comparisons with the Salvador I genome sequence reveal many DNA sequences absent from the reference genome sequence and numerous previously uncharacterized protein coding genes.

## Results/Discussion

### *De novo* genome assembly of a *Plasmodium vivax* field isolate

We selected a Cambodian *P. vivax* field isolate previously sequenced in our laboratory [6] to perform *de novo* assembly of large genomic regions. *De novo* assemblies are complicated when high levels of heterozygosity are present in the sequencing dataset. While *P. vivax* is haploid in humans, we have shown that most patients are infected by several genetically distinct parasites leading to many apparently heterozygous positions (with the additional caveat that the two alleles are often present in unequal proportions [6]). We therefore selected for our analysis massively parallel sequencing reads generated from a Cambodian patient (C127) in which more than 95% of all *P. vivax* DNA derived from a single strain. We hypothesized that this lower infection complexity would reduce apparent heterozygosity and facilitate *de novo* genome assembly. Despite residual contamination with human DNA, we have sequenced this sample at high coverage: on average, any given position of the *P. vivax* reference genome sequence was covered by more than 400 sequence reads (see [6] for details). Note that our previous genomic analyses [6] did not reveal any atypical features (for example the C127 fell within the range of *P. vivax* diversity) suggesting that this sample was not a particularly divergent strain, nor more closely related to a monkey malaria parasite (e.g., *P. cynomolgi* or *P. simiovale*).

We first identified and removed DNA sequences originating from the host genome by mapping all reads to the human genome reference sequence. This process is unlikely to accidentally remove many *P. vivax* DNA sequences: less than 0.01% of the assembled *P. vivax* reference genome sequence would be discarded by this procedure (2,220 DNA sequences of 100 bp can be mapped on the human genome out of 22,577,222 possible sequences). We then applied an error-correction algorithm to the filtered dataset and *de novo* assembled the short reads into large contiguous DNA sequences (contigs). After removing highly homologous contigs (n = 99), the final assembly contained 2,857 contigs larger than 1,000 bp, with a median size of 4,255 bp and a total assembly length of ∼28.4 million nucleotides (**Table 1**).

**Table 1:** *De novo* assembly summary statistics.

	# Contigs	N50^1^ (in bp)	N90^2^ (in bp)	Max (bp)	Median (bp)	Total length (bp)
All contigs	11,937	21,245	2,774	256,827	101	30,018,526
Contigs >1 kb	2,857	22,917	3,912	256,827	4,255	28,450,954

1 N50 is a statistic calculated such that 50% of the total length of the assembly is contained in contigs equal or larger than this value.

2 N90 is a statistic calculated such that 90% of the total length of the assembly is contained in contigs equal or larger than this value.

**Figure 1:**
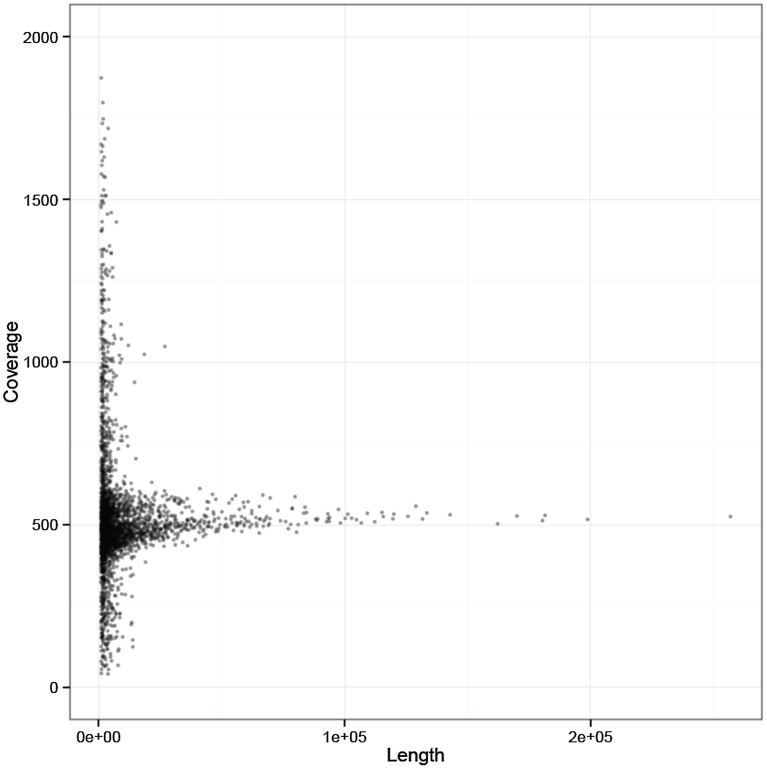
Sequence coverage for all *de novo* assembled contigs. The figure shows the average sequence coverage (y-axis, in read per base) of each *de novo* assembled C127 contig according to the contig length (x-axis, in bp). Six short (<200 bp) contigs displayed coverage greater than 2,000 reads by base and are not shown on this figure.

We mapped back all massively parallel sequencing reads generated from the C127 sample onto the *de novo* assembly and calculated the average sequence coverage of each contig. Consistent with our initial mapping on the *P. vivax* reference genome, we observed that most contigs were covered at ∼500× and therefore likely originated from the *P. vivax* genome (**Figure 1**). A minority of contigs, all shorter than 1 kb, showed significantly higher or lower coverage and possibly represented a combination of unfiltered host DNA sequences, *P. vivax* sequences from one of the minor strains present in C127, or repeated DNA sequences of the *P. vivax* genome.

It is important to note that the assembly described here is too fragmented to provide an alternative to the Salvador I reference genome sequence. However, the length of the DNA sequences assembled is sufficient to enable genomic analyses and identify DNA sequences absent from the Salvador I reference genome.

### Identification and characterization of novel *P. vivax* genomic sequences

We mapped all *de novo* assembled C127 contigs greater than 1 kb back to the *P. vivax* Salvador I reference genome sequence. 1,477 out of 2,857 assembled contigs mapped more than 95% of their length to the Salvador I reference genome sequence accounting for 21.6 Mb (or 95.6% of the assembled reference genome). For the rest of our analyses we focused on the 362 contigs that each contained more than 5 kb of contiguous DNA sequences absent from the Salvador I reference genome sequence (**Table S1**). In total, these contigs accounted for 3.8 Mb of novel *P. vivax* DNA sequence.

Fourteen of these 362 contigs contained at least 1 kb of DNA sequence mapped to the Salvador I reference sequence allowing us to position them on the genome, almost always in telomeric regions (**Table S2**). Telomeric regions are typically difficult to assemble into a final genome sequence as they contain low complexity- and repeated DNA sequences. To test whether the unmapped contigs may have been originally sequenced from the Salvador I strain but had not been successfully assembled in the published genome sequence, we compared their DNA sequences to the NCBI nucleotide collection database. Indeed, 85 contigs mapped to telomeric YAC sequences generated for sequencing the Salvador I genome and likely represented DNA sequences present in the Salvador I strain but unassembled in the final reference genome sequence (**Table S1**). To further investigate the genomic locations of the unmapped contigs, we mapped them to the *P. knowlesi* genome sequence [12], which is, for the most part, syntenic with the *P. vivax* genome [8]. Consistent with our hypothesis, we observed that the 59 of the unmapped C127 contigs mapped to the telomeric ends of *P. knowlesi* chromosomes (**Figure S1**).

We then applied a gene prediction algorithm [13] to identify putative protein coding sequences in the 362 unmapped contigs and compared the predicted protein sequences with sequences of well-annotated protein coding genes. Of the 362 unmapped contigs, 235 contained predicted protein coding genes most similar to a member of the *vir* multigene family. These genes are often located in the telomeric regions of the *P. vivax* genome and have been shown to exhibit tremendous DNA sequence diversity among samples [5]. This analysis confirmed that most DNA sequences assembled from C127 that did not map to the Salvador I reference genome originated from telomeric or sub-telomeric regions. Overall, we identified 792 novel *P. vivax* genes, including 366 vir genes, 35 members of other multigene families, 218 genes most similar to an annotated *Plasmodium* hypothetical protein and 13 predicted protein coding genes most similar to well-characterized *P. vivax* or P. *cynomolgi* genes (**Table 2**).

**Table 2:** Predicted genes in novel *P. vivax* DNA sequences.

Most similar gene in NCBI nr database	Number of predicted genes
**Total**	**792**
**Similar to an annotated ** ***Plasmodium*** ** gene** *	**654**
• Multigene families	
vir (*P. vivax*)/CYIR (*P. cynomolgi*) gene	366/22
other multigene family genes	35
• Hypothetical proteins	218
• Other genes	
Merozoite Surface protein 3 gamma (*P. vivax*)	1
Actin (*P. vivax*)	1
Casein kinase II beta (*P. vivax*)	1
Ribosomal S27a (*P. vivax*)	1
Proteosome subunit (*P. vivax*)	1
Subtelomeric transmembrane protein 1 – Pvstp1 (*P. vivax*)	1
*Plasmodium* subtelomeric A protein – PST-A (*P. vivax*)	1
Reticulocyte-binding protein 2e – RBP2e (*P. cynomolgi*)	5
Duffy-binding protein 2 – DBP2 (*P. cynomolgi*)	1

* best blast hit corresponds to a *Plasmodium* gene

### Analysis of *vir* multigene family in the C127 genome

**Figure 2:**
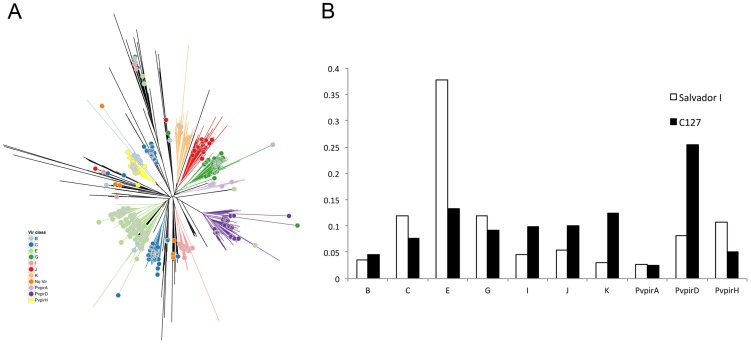
Analysis of *vir* genes in the C127 contigs. (**A**) Phylogenetic tree showing the relationships between the protein coding sequences of *vir* genes from the Salvador I reference genome (solid dots) and those predicted from the C127 contigs (branches without dots at the tips). Annotated *vir* genes (solid dots) are colored according to their subfamilies. Nodes used to assign predicted C127 *vir* genes into subfamilies are shown by the colored branches derived from them. (**B**) Proportion of genes assigned to each major *vir* subfamily for Salvador I (empty bars) and C127 (black bars).

Most of the *de novo* assembled contigs that did not map to the Salvador I reference genome contained one or more genes most similar to an annotated *vir* gene (**Table 2**). *vir* genes are thought to be involved in evasion of host adaptive immune response, cytoadherence and virulence; and have been previously shown to be extremely diverse among *P. vivax* parasites [5], [14]–[17]. *vir* genes predicted in the C127 contigs harbored the same structure as well-annotated vir genes, regardless of whether they are located in contigs mapping the reference genome or in novel sequences absent from the Salvador I genome (**Figure S2**). This observation suggests that most of the novel *vir* genes might be functional. *vir* genes are typically classified, based on sequence homology, into subfamilies [14], [18] that are regulated differently and might have different functions [18], [19]. We used our *vir* gene predictions and assigned each C127 *vir* gene into a given subfamily using a phylogenetic approach (**Figure 2A**) to test whether the composition of the *vir* gene repertoire differed between the Salvador I strain and C127. Our analyses revealed that the proportion of genes assigned to different *vir* subfamilies was indeed quite different between Salvador I and C127 (**Figure 2B**) and that these differences were not solely due to *vir* genes located in DNA sequences absent from the Salvador I genome (**Figure S3**). This suggests that, even at a given chromosomal locus, *vir* genes from different subfamilies may be present in different samples, perhaps due to rapid accumulation of mutations at these loci or to non-homologous recombination events. Overall, our analysis reinforces the notion that *vir* genes are extremely diverse, within and between *P. vivax* strains, and that our current catalogue of *vir* genes is likely to be far from complete.

### Identification of two novel *P. vivax* invasion protein genes

We identified two contigs without homologous DNA sequences in the Salvador I reference genome that contained predicted genes similar to *P. cynomolgi* invasion protein genes (**Table 2**).

**Figure 3:**
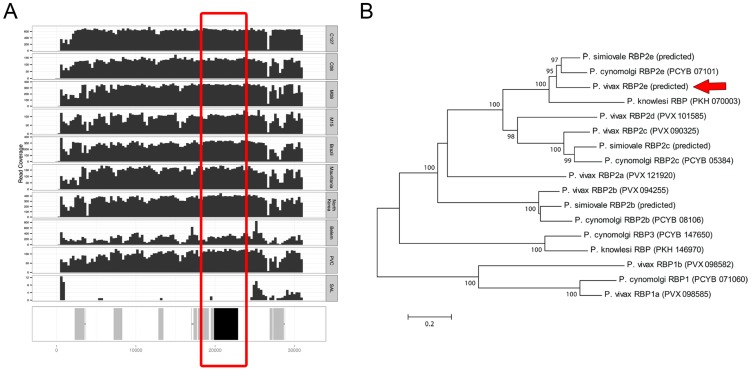
Novel *P. vivax* Reticulocyte-binding protein gene. (**A**) Next-generation sequencing read coverage along the ∼30 kb contig assembled from the C127 sample. The samples displayed are, from top to bottom: C127, C08, M08, M15, Brazil I, Mauritania I, North Korea, Belem, Chesson and Salvador I. The bottom track shows, in grey, predicted protein coding genes and in black, the position of the predicted reticulocyte-binding protein gene (also highlighted by the red box). Note that there is no coverage of the contig in Salvador I. (**B**) Phylogenetic tree showing the relationships among protein sequences of *P. vivax*, *P. cynomolgi*, *P. simiovale* and *P. knowlesi* RBP genes. The position of the predicted *P. vivax* RBP2e gene is highlighted by the red arrow.

**Figure 4:**
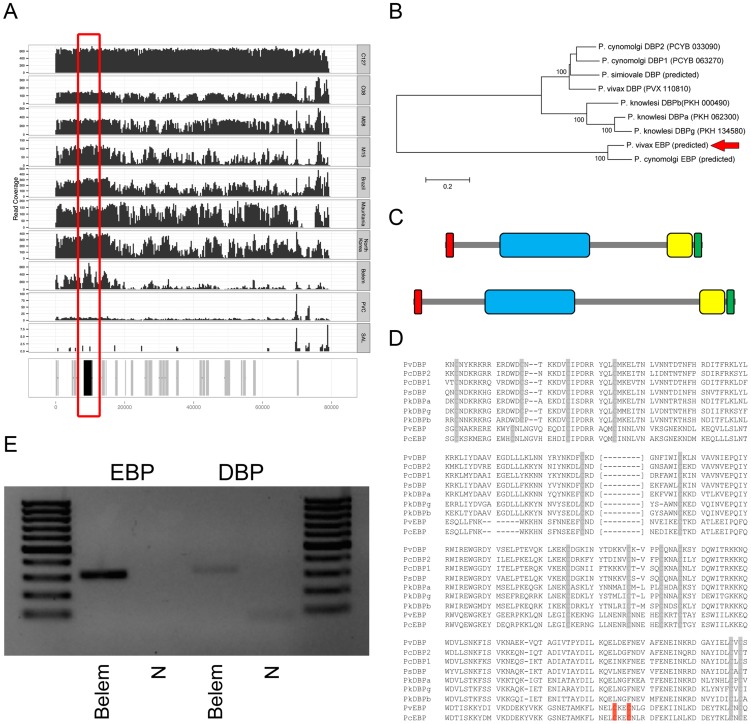
Novel *P. vivax* Erythrocyte-binding protein gene. (**A**) Sequence coverage along the 80 kb contig containing a novel predicted *P. vivax* Erythrocyte-Binding Protein gene. The bottom track shows, in grey, predicted protein coding genes and, in black, the position of the predicted EBP gene (also highlighted by the red box). The upper panels display next-generation sequencing read coverage along the contig sequence. The samples are from top to bottom: C127, C08, M08, M15, Brazil I, Mauritania I, North Korea, Belem, Chesson and Salvador I. Note that in the Salvador I sample, there are essentially no reads mapping to this contig. (**B**) Phylogenetic tree showing the relationships among EBP protein sequences from *P. vivax*, *P. cynomolgi*, *P. simiovale* and *P. knowlesi*. The position of the novel predicted *P. vivax* EBP gene is highlighted by the red arrow. (**C**) Comparison of the protein domain annotations for the novel predicted EBP gene (top) and the known *P. vivax* DBP gene. The red box indicates the signal sequence; the blue box the Duffy-binding like domain; the yellow box the C-terminus cysteine-rich domain and the green box the transmembrane domain. (**D**) Amino acid alignment of the Duffy-binding like domain for different *Plasmodium* DBP genes and the novel *P. vivax* and *P. cynomolgi* EBP genes. The alignment shows the amino acid positions 215–509 of PvDBP (the section of the alignment not displayed corresponds to amino acid 303–371). The grey boxes indicate conserved cysteine positions. The red boxes indicate the positions of two additional cysteines present in the novel EBP genes. (**E**) The novel *P. vivax* EBP gene is expressed in blood-stage parasites. The gel picture shows the PCR products for the novel EBP gene (left) and known DBP gene (right) amplified from cDNA of the Belem strain. Note that, for both genes, the primers are located on either side of an intron and that the product size is consistent with amplification of cDNA molecules and excludes DNA contamination.

A 31 kb contig (**Figure 3A**) contained 10 predicted protein coding genes including one single-exon gene of 3,003 coding nucleotides (encoding 1,001 amino acids) that displayed 74% identity over 99% of its amino acid sequence to the *P. cynomolgi* RBP2e gene. An 80 kb contig (**Figure 4A**) contained 20 predicted genes including eight *vir* genes and six genes most similar to *P. cynomolgi* hypothetical proteins. One of the remaining predicted genes contained 2,409 coding nucleotides (encoding 803 amino acids) in two exons. When compared to all annotated protein sequences present in the NCBI database, this predicted protein coding sequence was most similar to the *P. cynomolgi* DBP2 gene but with only 37% identity along 67% of its amino acid sequence.

To test whether these DNA sequences were present in *P. vivax* parasites other than C127, we mapped on these contigs reads generated from samples that we [6] and others [5] have previously sequenced. Interestingly, while we observed no read coverage for these contigs in the Salvador I sample that we re-sequenced, all other samples tested showed relatively uniform coverage along these entire contig sequences (**Figure 3A** and **Figure 4A**). These results indicated that these novel DNA sequences were indeed absent from the Salvador I genome but present in all other *P. vivax* genomes sequenced so far. We validated these results by locus-specific PCRs that failed to amplify Salvador I DNA but, under the same conditions, successfully amplified DNA from other *P. vivax* samples (**Figure S4**). Note that we detected these contig sequences in several monkey-adapted strains suggesting that these genes were not systematically deleted from the *P. vivax* genome during host switch. In fact, mapping next-generation sequencing reads generated from various monkey-adapted strains on all contigs assembled from the C127 sample revealed that none of the novel DNA sequences were systematically absent from monkey-adapted stains. This observation suggests that genomic adaptations to the novel host (if any) may not involve deletions of large chromosomal segments.

### PvRBP2e is deleted in the Salvador I strain but is present in other *P. vivax* samples

To further examine the putative *P. vivax* RBP gene, we compared its predicted amino acid sequence with sequences of known *P. vivax* [8], *P. cynomolgi* [10] and *P. knowlesi* [12] RBP genes as well as homologous sequences predicted from the genome assembly of the closely related species *P. simiovale*. The novel *P. vivax* RBP gene clustered within the RBP2 gene family and showed highest sequence similarity to the *P. cynomolgi* RBP2e gene and to a predicted *P. simiovale* gene (**Figure 3B**). In this regard, it is interesting to note that sequencing and annotation of the *P. cynomolgi* genome [10] showed the presence of the RBP2e gene in three different *P. cynomolgi* strains as well as in the *P. knowlesi* genome but failed to identify a *P. vivax* orthologous gene in the Salvador I genome. Our analyses suggested that RBP2e was actually present in most *P. vivax* parasites but absent in the Salvador I strain.

The similarity between the C127, the *P. cynomolgi* and *P. simiovale* DNA sequences extended far beyond the RBP2e protein coding gene sequence and could be detected for several kb outside the gene boundaries (**Figure S5**). This synteny confirmed that the RBP2e genes of the different *Plasmodium* species were indeed orthologous genes. In addition, using DNA sequence similarity to the *P. cynomolgi* DNA sequence, we were able to determine that the *P. vivax* RBP2e gene was likely located at the telomeric end of chromosome 7 (similar to its position in the *P. cynomolgi* genome), approximately 25 kb upstream of the annotated start of the Salvador I chromosome 7 (**Figure S5**).

One puzzling observation was that the predicted RBP2e gene was significantly shorter in *P. vivax* than in other *Plasmodium* species: the predicted *P. vivax* protein was only 1001 amino acid long, compared to 2732, 2745 and 2927 amino acids in, respectively, *P. cynomolgi*, *P. simiovale* and *P. knowlesi*. In addition, the C127 contig contained four additional predicted genes with short protein coding sequences and located immediately next to the predicted *P. vivax* RBP2e, which also showed high similarity with *P. cynomolgi* RBP2e (**Table 2** and **Figure S6A**). Close inspection of the DNA sequence alignments between the C127 contig and *P. cynomolgi*, *P. simiovale* and *P. knowlesi* sequences revealed several short out-of-frame deletions in the *P. vivax* DNA sequence that occurred between the beginning of the protein sequence predicted in the other *Plasmodium* genomes and the beginning of the protein sequence predicted in *P. vivax*, resulting in the introduction of premature stop codons and multiple short predicted protein coding sequences (**Figure S6A**). To test whether the deletions in the C127 contigs were genuine or artifacts of the *de novo* assembly, we mapped sequencing reads from C127 and other *P. vivax* samples onto the assembled C127 contig sequence. This analysis confirmed the accuracy of the contig DNA sequence and indicated that other *P. vivax* samples also carried deletions resulting in shorter forms of the RBP2e protein coding gene. Amplification by locus-specific PCRs of *P. vivax* cDNA showed that the RBP2e was expressed by blood-stage *P. vivax* parasites. However, this analysis also revealed a single extended transcript that encompassed several (if not all) of the *P. vivax* short predicted protein coding genes, similar to the RBP2e gene structure predicted in *P. cynomolgi*, *P. simiovale* and *P. knowlesi* (**Figure S6B**). Sanger sequencing of the amplified cDNA products confirmed the premature stop codons observed in the C127 contig sequence. One possible explanation for these observations was that the RBP2e protein coding sequence had been disrupted by several deletions in *P. vivax*, resulting in a non-functional protein, while the proximal promoter and other regulatory elements were kept intact and could still induce transcription of this probable pseudogene. However it is important to note that PfRH5, a gene encoding a *P. falciparum* RBP, shows a similar pattern, with an early stop codon compared to other PfRH genes, but is translated into a functional protein that binds specifically to human erythrocytes [20]. Further studies will therefore be required to rigorously determine whether RBP2e is functional in *P. vivax*.

### A novel *P. vivax* Erythrocyte-Binding Protein gene sequence

We next examined the novel predicted *P. vivax* gene displaying similarities with the *P. cynomolgi* DBP2. We first compared its predicted protein coding sequence with the sequences of known *P. vivax*, *P. cynomolgi* and *P. knowlesi* DBP genes as well as predicted homologous protein sequences. Phylogenetic analyses revealed that the novel predicted gene fell outside the cluster of known *Plasmodium* DBPs which included *P. vivax* DBP, two *P. cynomolgi* genes, one *P. simiovale* and three *P. knowlesi* genes. The putative novel protein coding sequence was only similar to a previously unannotated protein coding gene predicted in the *P. cynomolgi* genome (with 83% identity at the amino acid level) (**Figure 4B**). Interestingly, this DNA sequence similarity extended far beyond the predicted protein coding sequence since the contigs containing the novel *P. vivax* and *P. cynomolgi* predicted genes could be aligned over more than 12 kb (**Figure S7**) suggesting that these genes were indeed orthologous. Unfortunately, the lack of overlapping DNA sequences between either of these contigs and any of the *Plasmodium* genomes assembled prevented us from determining the position of this predicted gene in the *P. vivax* genome.

While the novel predicted *P. vivax* gene clearly differed from known DBP genes, it displayed several interesting features. The predicted protein sequence contained a N-terminal 22 amino acid long signal peptide, a 276 amino acid Duffy-binding like domain (DBL or pfam05424), partially overlapping with a *P. falciparum* erythrocyte membrane protein 1 (PfEMP1) domain, an 81 amino acid erythrocyte binding antigen 175 (EBA-175) domain, also referred to as a C-terminal cysteine-rich domain [21], [22], and a final 20 amino acid long transmembrane domain (**Figure 4C**). These features are the hallmark of the Erythrocyte-Binding Protein (EBP) superfamily [21], which includes the DBPs and *P. falciparum* EBA proteins, and are critical for invasion of human RBC by malaria parasites. The novel *P. vivax* predicted EBP gene contained a single DBL domain, as in the *P. vivax*, *P. knowlesi* and *P. cynomolgi* DBPs, while all *P. falciparum* EBP genes carry two consecutive domains [21] (note that RBPs have a different structure and do not harbor a DBL domain). The DBL domain is classically characterized by 12 conserved cysteines, five of them (cysteines 4 to 8) being critical for binding to erythrocyte ligands [23], [24]. Alignment of the novel predicted *P. vivax* and *P. cynomolgi* EBP amino acid sequences with known *Plasmodium* DBPs revealed that, while the rest of the protein sequence showed numerous differences, 11 out of the 12 cysteines of the DBL domain were conserved, including all cysteines of the minimal region implicated for receptor recognition (**Figure 4D**). This observation suggested that the novel *P. vivax* predicted EBP might also be able to bind to human erythrocytes. In the C-terminus cysteine-rich domain, 6 out of 8 cysteine positions were also conserved between this newly identified *P. vivax* EBP and known DBPs (**Figure S8**) indicating that this domain might, as in other *Plasmodium* EBPs [25], [26], regulate the export of the protein to the micronemes where it can be displayed at the apical end of the merozoites during erythrocyte invasion.

We then investigated the level of conservation of the predicted EBP coding sequence among *P. vivax* samples. For ten sequenced *P. vivax* samples, sampled in Asia, Southwest Pacific, Africa and Central and South-America, in which most of *P. vivax* DNA derived from a single strain (see also [6]), we determined the predicted coding sequence directly from the DNA sequences of massively parallel sequencing reads. We did not observe any nonsense or frameshift mutations and the novel *P. vivax* EBP gene displayed only 11 polymorphic sites in 2,412 bp, including 2 synonymous- and 9 non-synonymous SNPs. For comparison, in the same samples the annotated *P. vivax* DBP gene displayed 32 polymorphic sites in 3,213 positions (4 synonymous and 28 non-synonymous SNPs) (**Figure S9**). Overall, the pattern of diversity observed among *P. vivax* samples showed no evidence that the novel predicted EBP gene was a pseudogene. Finally, we amplified cDNA generated from the Belem strain and showed that this novel EBP gene was expressed in blood-stage parasites (**Figure 4E**).

### Conclusion

By *de novo* assembly of a field isolate genome, we have shown that large genomic regions of the *P. vivax* genome had not been previously characterized because they were missing from the Salvador I strain used for generating the *P. vivax* reference genome. In total, we identified 3.8 Mb of novel DNA sequences containing close to 800 novel predicted genes. These included at least two putative invasion genes that had not been previously described in *P. vivax* and is consistent with observations in *P. falciparum* and *P. cynomolgi* that invasion genes are frequently deleted among strains [10], [27]–[29]. In particular, we described a novel predicted protein coding gene containing a DBL domain and a C-terminus cysteine-rich domain. While clearly different from all previously characterized *Plasmodium* DBPs, this predicted protein harbored all the key features of EBPs, suggesting that it might be able to bind to human erythrocytes and contribute to RBC invasion. This putative EBP gene is missing from the Salvador I strain but present in all other *P. vivax* strains tested, representing a wide geographical range, and is expressed in blood-stage parasites. While further studies will be required to determine whether this gene is functional and characterize its role in erythrocyte invasion, it is tempting to speculate that the novel *P. vivax* EBP may enable interactions with erythrocyte membrane proteins other than the Duffy antigen and contribute to alternative invasion pathways. In this regard, it is important to note that, so far, *in vitro* studies have consistently shown that PvDBP is unable to bind Duffy-negative erythrocytes, suggesting that its only ligand is the human Duffy antigen. Recent observations of *P. vivax* infections of Duffy-negative individuals [30]–[33] therefore remain mysterious. The discovery of this second *P. vivax* EBP gene provides a candidate to possibly explain this alternative invasion mechanism and could perhaps constitute a first step towards understanding the molecular bases underlying *P. vivax* infection of Duffy-negative individuals. Overall, our study showed that genomic analyses of *P. vivax* parasites can complement *in vitro* approaches and significantly contribute to improving our understanding of *P. vivax* biology.

## Materials and Methods

### *De novo* assembly of a Cambodian *P. vivax* field isolate

For *de novo* genome assembly, we focused on massively parallel sequencing data generated previously from a Cambodian *P. vivax* field isolate (C127) [6]. We first removed host DNA sequences by mapping all 211,061,945 paired-end reads generated from this sample on the human reference genome sequence [34] using Bowtie 2 [35] and low-stringency mapping criteria (using the very-sensitive parameter). This approach removed 13,214,696 read pairs (6%). Next, we applied the algorithm implemented in Quake [36] to correct sequencing errors and remove low-quality reads using a k-mer of 17 nucleotides. We then generated a *de novo* assembly from the remaining 161,300,780 corrected read pairs using ABySS [37] and a k-mer of 51 bp (we determined the optimal k-mer by testing different k values on a subset of 24,730,906 paired-end reads and comparing the resulting assemblies). Finally, we mapped all contigs greater than 1 kb onto each other using Lastz [38] and, when two contigs were more than 95% identical over more than 95% of their length, we discarded the shorter sequence to filter out highly homologous contigs.

We then mapped all C127 paired-end reads on the final assembly using Bowtie 2 and calculated the average read coverage of each contig (excluding 100 bp at the end of each sequence to avoid mapping biases). We also mapped reads generated from other *P. vivax* samples that we [6] and others [5] have analyzed to the C127 final contigs to determine sequence coverage in each sample and identify DNA polymorphisms. We included in these analyses: two additional Cambodian field isolates (C08 and C15), three Malagasy field isolates (M08, M19 and M15) and six monkey-adapted strains (Salvador I, Belem, Chesson, Brazil I, North Korea and Mauritania I).

### Mapping of C127 contigs to the Salvador I reference genome and identification of novel DNA sequences

To identify *P. vivax* DNA sequences missing from the Salvador I genome [8], we mapped all contigs to the reference genome sequence using Lastz, allowing for up to 10% DNA sequence divergence. We then screened for contigs in which more than 5 kb of contiguous DNA sequence did not map the *P. vivax* reference genome sequence. We then compared these contig sequences to the NCBI nucleotide database (nt) using blast to identify loci that had been originally sequenced from the Salvador I strain but were not included in the final genome assembly.

### Gene predictions

For all contigs with more than 5 kb of contiguous DNA sequence that did not map to the Salvador I reference genome, we searched for putative protein coding gene sequences using the SNAP gene prediction algorithm [13]. We used the NCBI gene annotations of the *P. vivax* Salvador I genome as the training set for the parameter estimation of the hidden Markov model. We then compared each predicted protein coding gene sequence to annotated proteins deposited in the NCBI non-redundant protein database (nr) to preliminarily characterize these putative protein coding genes.

### *vir* gene analysis

To optimize gene predictions for the members of the *vir* multigene family, we re-ran the SNAP algorithm on all *de novo* assembled contigs but using only *vir* genes annotated from the Salvador I reference as a training set. We translated the predicted genes into proteins and used blastp against the NCBI nr database to identify which predicted genes were most similar to *vir* genes. We then used Clustal Omega to align the protein sequences of the predicted *vir* genes with those of all annotated *vir* genes from the Salvador I genome and reconstructed a phylogenetic tree using FastTree. Finally, we annotated the branch of this tree according to the known *vir* subfamily annotations [18] and determined which node best defined each major *vir* subfamily using the following criteria: i) branches from this node must account for as many *vir* genes of the subfamily as possible, ii) branches derived from the node should include less than three *vir* gene from other subfamilies, iii) the node must be supported by a bootstrap value greater than 90% and iv) when multiple nodes fulfilled these previous conditions, the node closest to the root was selected. We then considered that all predicted *vir* genes downstream of one of these defining nodes belonged to the corresponding *vir* sub-families.

### Experimental validation

To validate the novel invasion protein coding genes and exclude the possibility that their DNA sequences resulted from assembly artifacts, we designed locus-specific primers and directly amplified these genes by PCR from genomic DNA. A putative *P. vivax* RBP gene was amplified using the primers 5′- AGAAGCTCTGGAGACACAAGC-3′ and 5′- GGTTCGTCCCTTTTCACCGT-3′ and a putative *P. vivax* EBP gene was amplified using the primers 5′-CAGCACAAAGACGACGGGTA-3′ and 5′-TCGTCTTTCTTTTTCGTCCTGC3′. We used the following PCR conditions: 94C for 3 min, followed by 40 cycles of 94°C for 60 s, 56°C for 45 s, and 72°C for 2 min. We sequenced all PCR products using Sanger chemistry and confirmed that the DNA sequences amplified were identical to the novel contig sequences.

To test whether these putative genes were transcribed in blood-stage parasites, we extracted total RNA from 150 µl of blood from a *Saimiri boliviensis* monkey infected with the Belem strain (30,000 parasites per µl) using standard Trizol/Chloroform protocol. After DNase I treatment, we prepared double-stranded cDNA using random hexamers and the Invitrogen SuperScriptIII Reverse Transcriptase kit. We then amplified putative genes using primers designed to span an intron and sequenced the resulting PCR products by Sanger sequencing. The novel *P. vivax* EBP transcript was amplified using the primers 5′-ATGGCAGCAGAAGGACTGAG-3′ and 5′-GCGGCGAGCACAATGAATAAT-3′. The novel *P. vivax* RBP transcript was amplified by several primer pairs described in Figure S6. We also amplified the *P. vivax* DBP transcript as a positive control using the primers 5′-AGGACATGACAGGGATAGCA-3′ and 5′- CAACAGCAGTATCAGCAACGC-3′.

### Identification of orthologous Duffy- and Reticulocyte-binding protein genes in other *Plasmodium* genomes

We used the assembled genome sequences and gene annotations generated respectively from the *P. knowlesi* H strain [12] and *P. cynomolgi* B strain [10]. We also generated 213,543,894 paired-end reads of 100 bp from DNA extracted from the *P. simiovale* strain maintained at the Centers for Disease Control and Prevention and *de novo* assembled this genome using the approach described above.

We retrieved the coding sequences from the *P. vivax* DBP and all annotated *P. vivax* RBPs from plasmodb [39] and added them to the sequences of the two novel predicted protein coding gene sequences identified in the C127 assembly. We used blast to search for similar protein coding sequences in the *P. simiovale*, *P. cynomolgi* and *P. knowlesi* genome assemblies. To avoid biasing the gene annotations towards the *P. vivax* annotations, we then used SNAP to predict protein coding gene sequences in any DNA sequence aligning to a *P. vivax* RBP or DBP gene sequence and translated these predicted genes into amino acid sequences.

### Protein domain annotations

We preliminarily annotated the putative *P. vivax* EBP gene using the NCBI conserved domains and protein classification tool [40] as well as the TMHMM algorithm [41] for identifying transmembrane domains and SignalP [42] for signal peptide.

### Sequence alignments and reconstruction of phylogenetic trees

We aligned DNA and protein sequences using Clustal omega [43] and reconstructed neighbor-joining trees with MEGA [44] using either the total number of amino acid differences or Jones-Taylor-Thornton [45] distances. Both methods yielded similar results in all reconstructions.

## Supporting Information

Figure S1 Distribution of the C127 contigs according to their position on the *P. knowlesi* genome. The figure displays, on each bottom row, the chromosomal (y-axis) and nucleotide location (x-axis, in bp) on the *P. knowlesi* genome of C127 contigs mapped to Salvador I (in grey) and 59 contigs that do not map to Salvador I (in black). For comparison, the top row shows, for each chromosome, the mapping of Salvador I DNA sequences onto the *P. knowlesi* genome.M stands for the mitochondrial genome.
(TIF)

Figure S2 Proportion of annotated (Salvador I, in red) or predicted (C127, in grey or black) *vir* genes (y-axis) according to their number of exons (x-axis). For C127, the *vir* genes are divided according to their location on contigs that map the reference genome (in black) or were absent from the reference genome (in grey).
(TIF)

Figure S3 Proportion of genes assigned to each major *vir* subfamily for Salvador I (red bars), C127 contig mapped on the reference genome (black bars) or *vir* gene located on novel DNA sequences (in grey).
(TIF)

Figure S4 Validation by locus-specific PCR. The gel shows the amplification products of the predicted EBP gene (left) and RBP gene (right) from genomic DNA extracted from three *P. vivax* samples (Salvador I, Chesson and C127) and a negative control (N).
(TIF)

Figure S5 Schematic DNA sequence alignment of the contigs generated from C127 (in red), *P. cynomolgi* (in blue) and *P. simiovale* (in green) and the *P. vivax* reference genome (in black). The grey box indicates the position of the RBP2e gene. Note that the *P. vivax* chromosome 7 sequence only starts several kb after the position of the predicted RBP2e gene.
(TIF)

Figure S6 (**A**) Schematic representation of the predicted RBP2e proteins in different *Plasmodium* genomes. The solid blue boxes represent the predicted protein coding sequence in, from top to bottom, *P. knowlesi*, *P. cynomolgi*, *P. simiovale* and *P. vivax* (C127). The black asterisks indicate the locations of out-of-frame deletions that, in *P. vivax*, introduce stop codons (vertical red bars) and lead to multiple short predicted proteins. The black arrows indicate the primers used to amplify cDNA from the Belem strain (from left to right, RBPi, RBP_3–4_ and RBP_4–5_). (**B**) The gel picture shows the PCR products obtained for the *P. vivax* RBP2e gene using cDNA generated from the Belem strain. The leftmost amplification (RBPo) targets a region upstream of the predicted RBP2e transcript and fails to yield any PCR product. The next three amplifications all yield PCR products of the correct size and identical to the contig sequence after Sanger sequencing and correspond to i) amplification spanning an intron in the first predicted PvRBP2e gene (RBPi), ii) amplification between the 3^rd^ and 4^th^ predicted PvRBP2e genes (RBP_3–4_) and iii) amplification between the 4^th^ and the 5^th^ predicted PvRBP2e genes (RBP_4–5_). The primer sequences used are as follow: RBPoF 5′-CCTCTTCTAGCTATTGAACTCACCA-3′, RBPoR 5′-AGCGTGCATGGCTAATTGTA-3′, RBPiF 5′-TGTGATCTTTTGTAACCTCTTGTTT-3′, RBPiR 5′-TTCCCAAGGAAGTGGCATGT-3′, RBP_3–4_F 5′-TCCTGAGACGGTGGATAACA-3′, RBP_3–4_R 5′-TGTTCTTTAGCTGTGTGAGACT-3′, RBP_4–5_F 5-GGACTACGAGCAAAGTGCAG-3′, RBP_4–5_R 5′-AGCGGATTCTTTGTGACTCCTT-3′.
(TIF)

Figure S7 DNA sequence identity (y-axis, in %) between the C127 contig carrying the predicted EBP gene (indicated by the red box) and its orthologous *P. cynomolgi* contig (x-axis, in bp).
(TIF)

Figure S8 Amino acid alignment of the C-terminus cysteine-rich like domain for DBP genes and the novel *P. vivax* and *P. cynomolgi* EBP genes. The grey boxes indicate conserved cysteine positions.
(TIF)

Figure S9 Phylogenetic tree showing the relationships between the amino-acid sequences of *P. vivax* samples for several RBP2 genes and the putative RBP2e.
(TIF)

Table S1 Contigs generated from the C127 sample with their length, percentage of the contig sequence mapped to the reference genome (%Sal I), number of predicted genes and blast comparisons to the nucleotide (nt) and protein (nr) databases.
(XLS)

Table S2 List of all contigs mapping partially to the Salvador I reference genome.
(DOC)
