# Supplementary figures and images for: De Novo Assembly of a Field Isolate Genome Reveals Novel Plasmodium vivax Erythrocyte Invasion Genes
Source: PLoS Negl Trop Dis. 2013 Dec 5;7(12):e2569. doi: 10.1371/journal.pntd.0002569 (PMC3854868; doi:10.1371/journal.pntd.0002569)

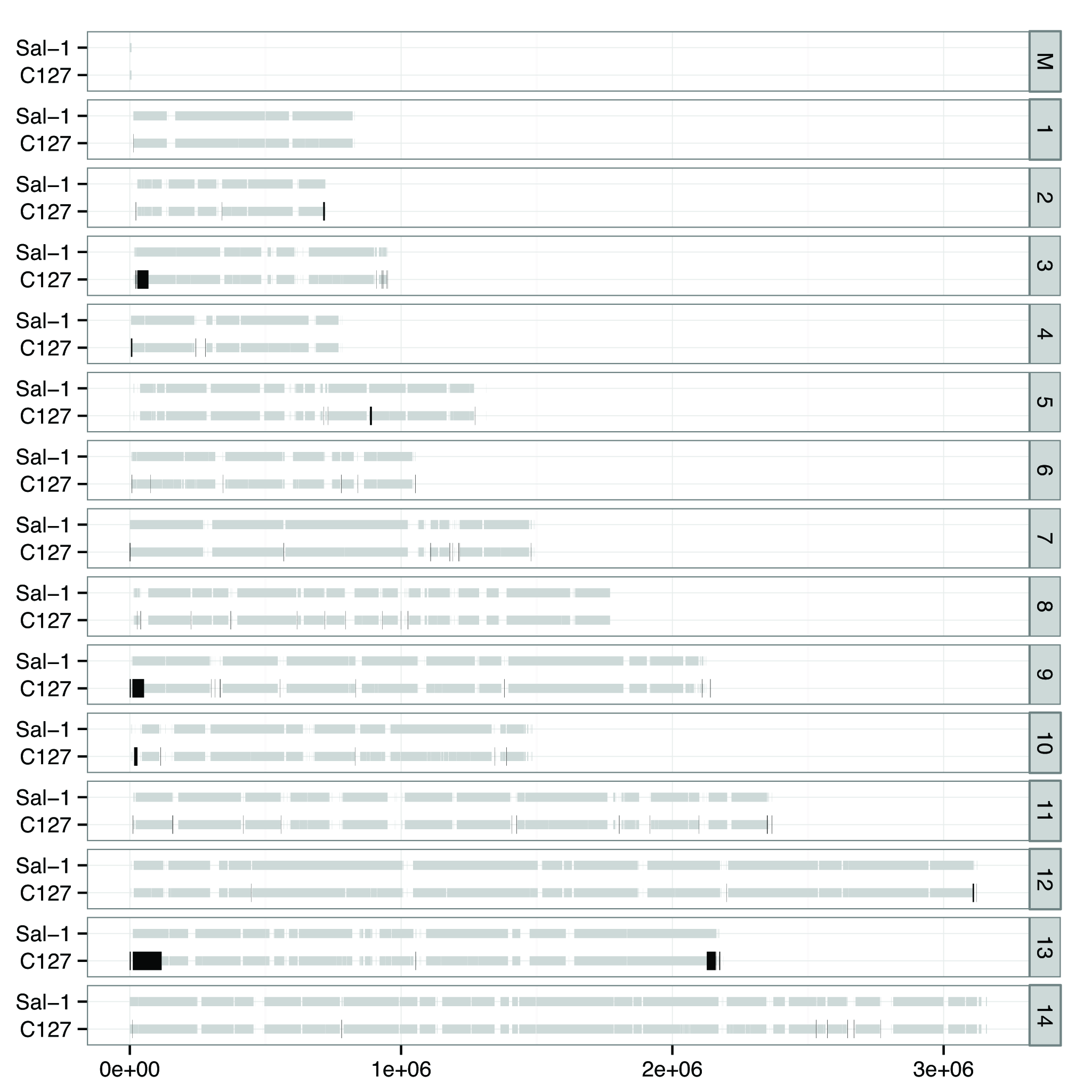

Supplement: Figure S1 — Distribution of the C127 contigs according to their position on the P. knowlesi genome. The figure displays, on each bottom row, the chromosomal (y-axis) and nucleotide location (x-axis, in bp) on the P. knowlesi genome of C127 contigs mapped to Salvador I (in grey) and 59 contigs that do not map to Salvador I (in black). For comparison, the top row shows, for each chromosome, the mapping of Salvador I DNA sequences onto the P. knowlesi genome.M stands for the mitochondrial genome. (TIF) [file pntd.0002569.s001.tif]

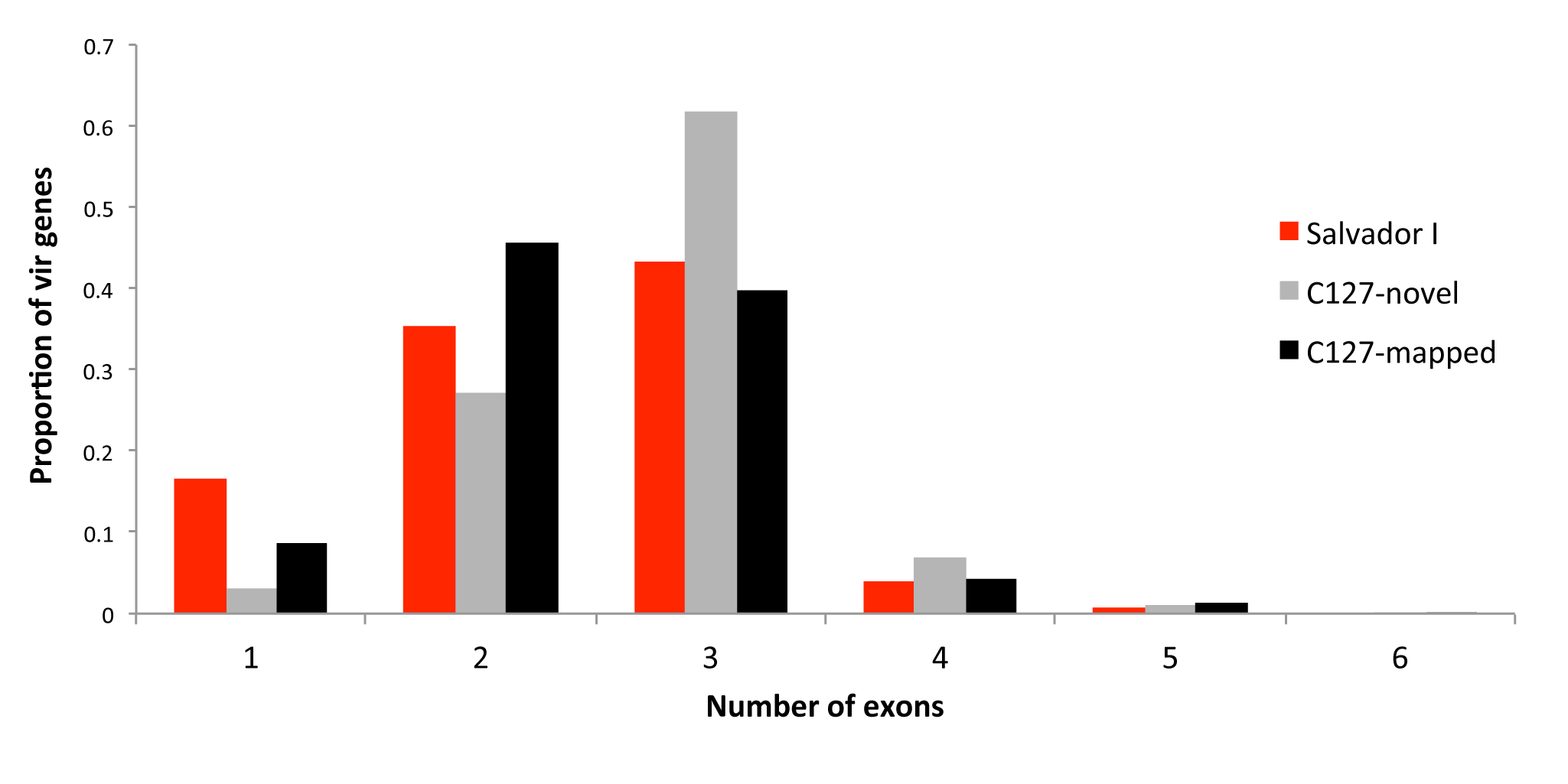

Supplement: Figure S2 — Proportion of annotated (Salvador I, in red) or predicted (C127, in grey or black) vir genes (y-axis) according to their number of exons (x-axis). For C127, the vir genes are divided according to their location on contigs that map the reference genome (in black) or were absent from the reference genome (in grey). (TIF) [file pntd.0002569.s002.tif]

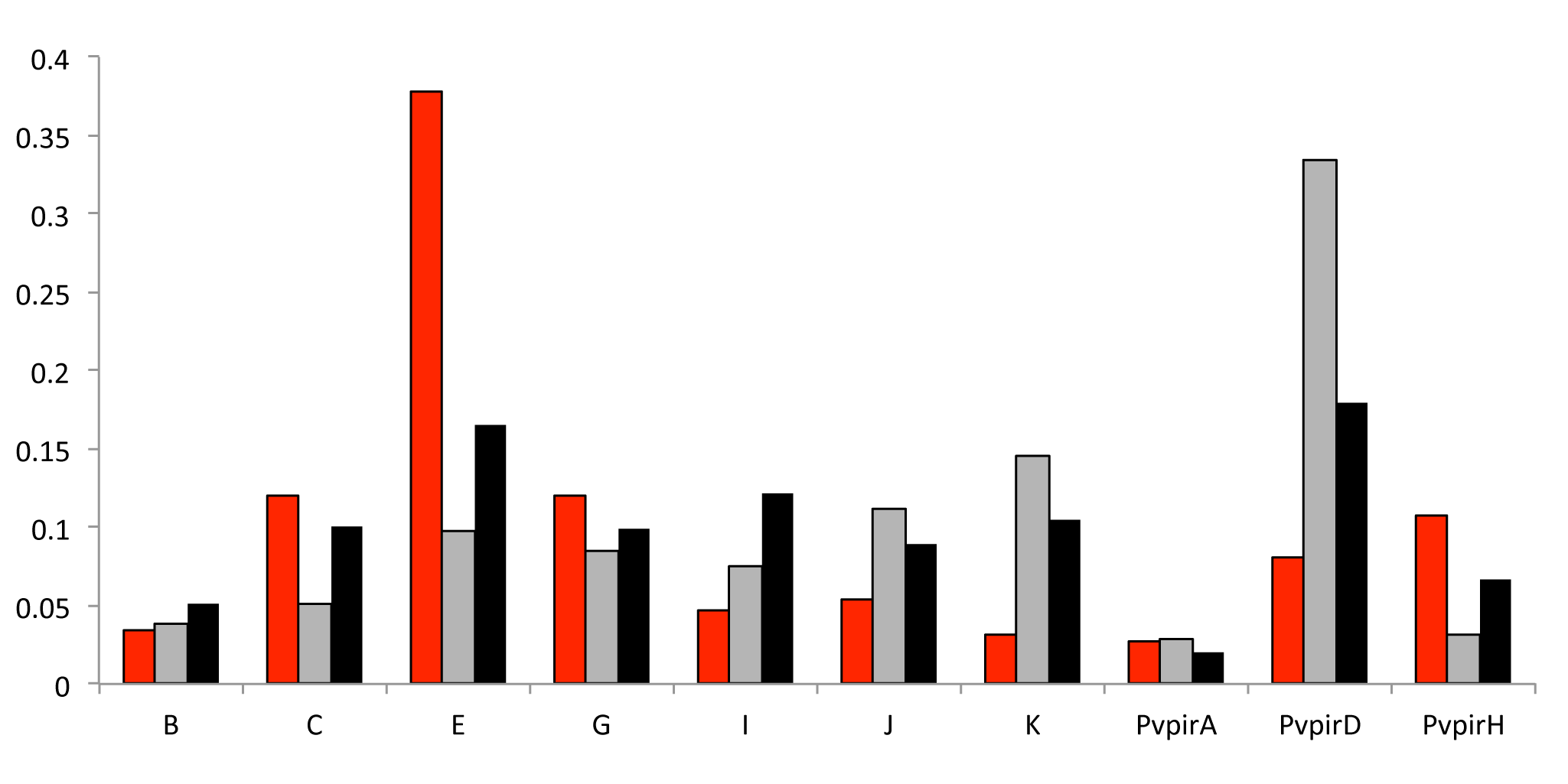

Supplement: Figure S3 — Proportion of genes assigned to each major vir subfamily for Salvador I (red bars), C127 contig mapped on the reference genome (black bars) or vir gene located on novel DNA sequences (in grey). (TIF) [file pntd.0002569.s003.tif]

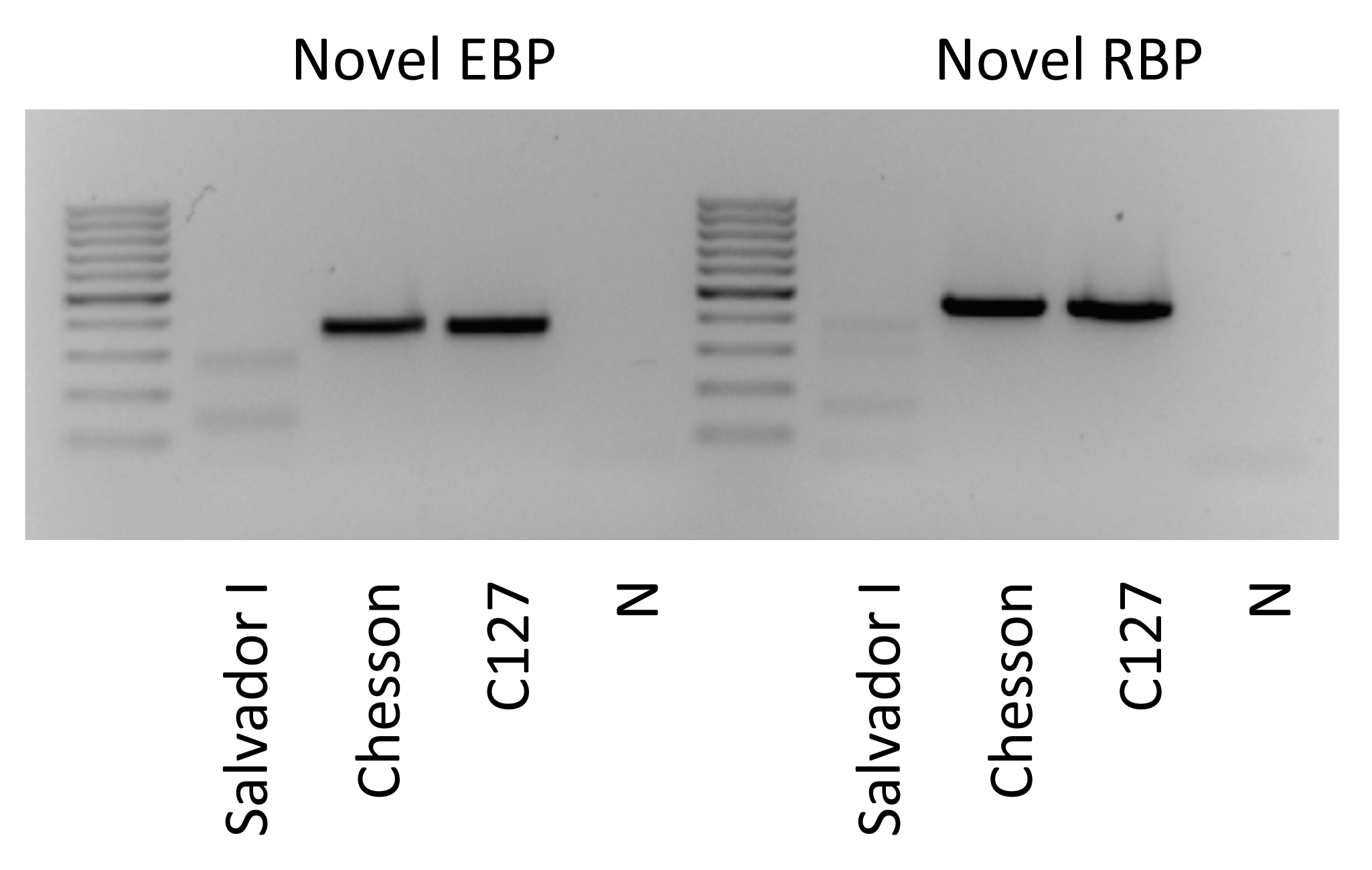

Supplement: Figure S4 — Validation by locus-specific PCR. The gel shows the amplification products of the predicted EBP gene (left) and RBP gene (right) from genomic DNA extracted from three P. vivax samples (Salvador I, Chesson and C127) and a negative control (N). (TIF) [file pntd.0002569.s004.tif]

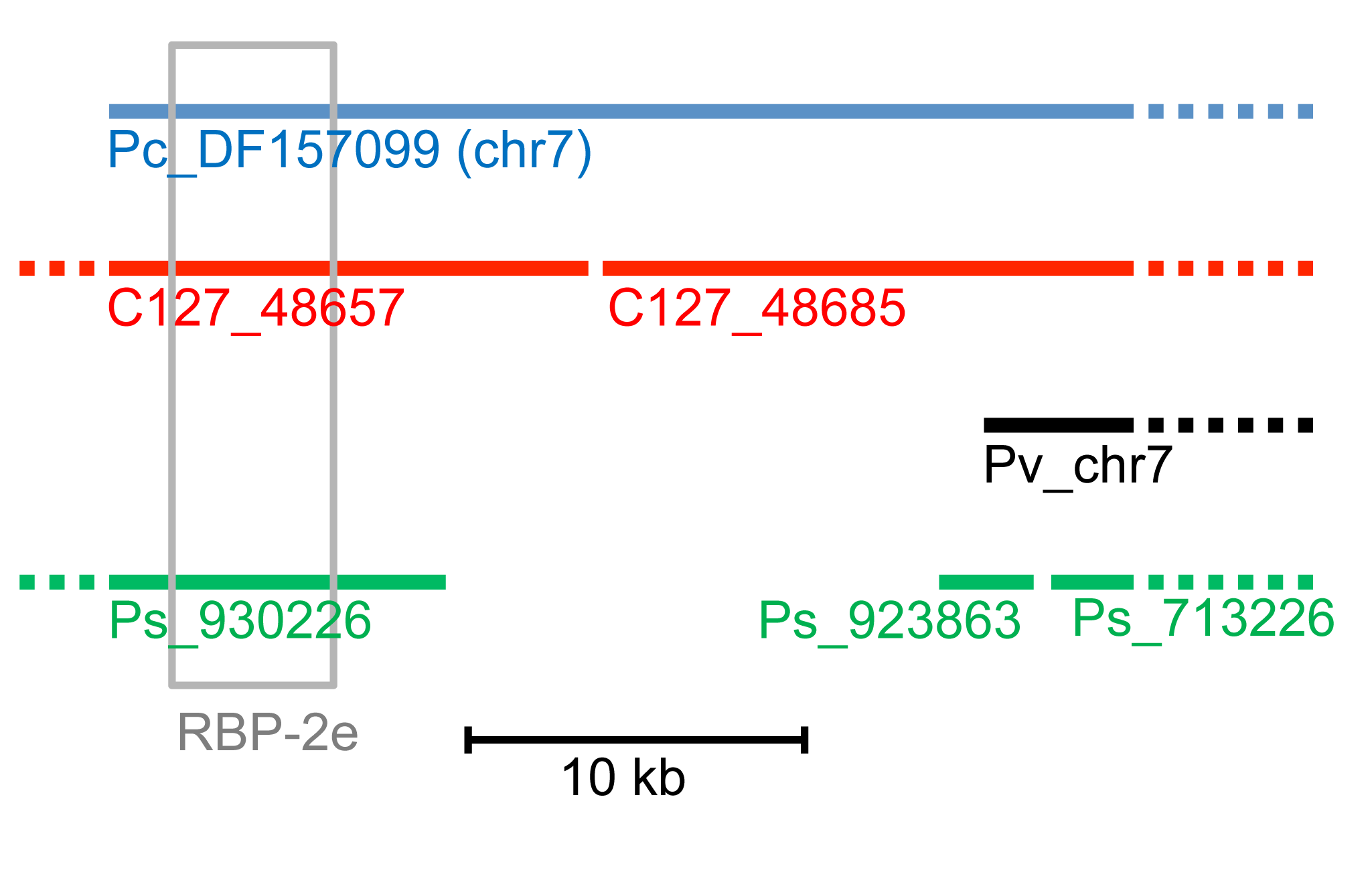

Supplement: Figure S5 — Schematic DNA sequence alignment of the contigs generated from C127 (in red), P. cynomolgi (in blue) and P. simiovale (in green) and the P. vivax reference genome (in black). The grey box indicates the position of the RBP2e gene. Note that the P. vivax chromosome 7 sequence only starts several kb after the position of the predicted RBP2e gene. (TIF) [file pntd.0002569.s005.tif]

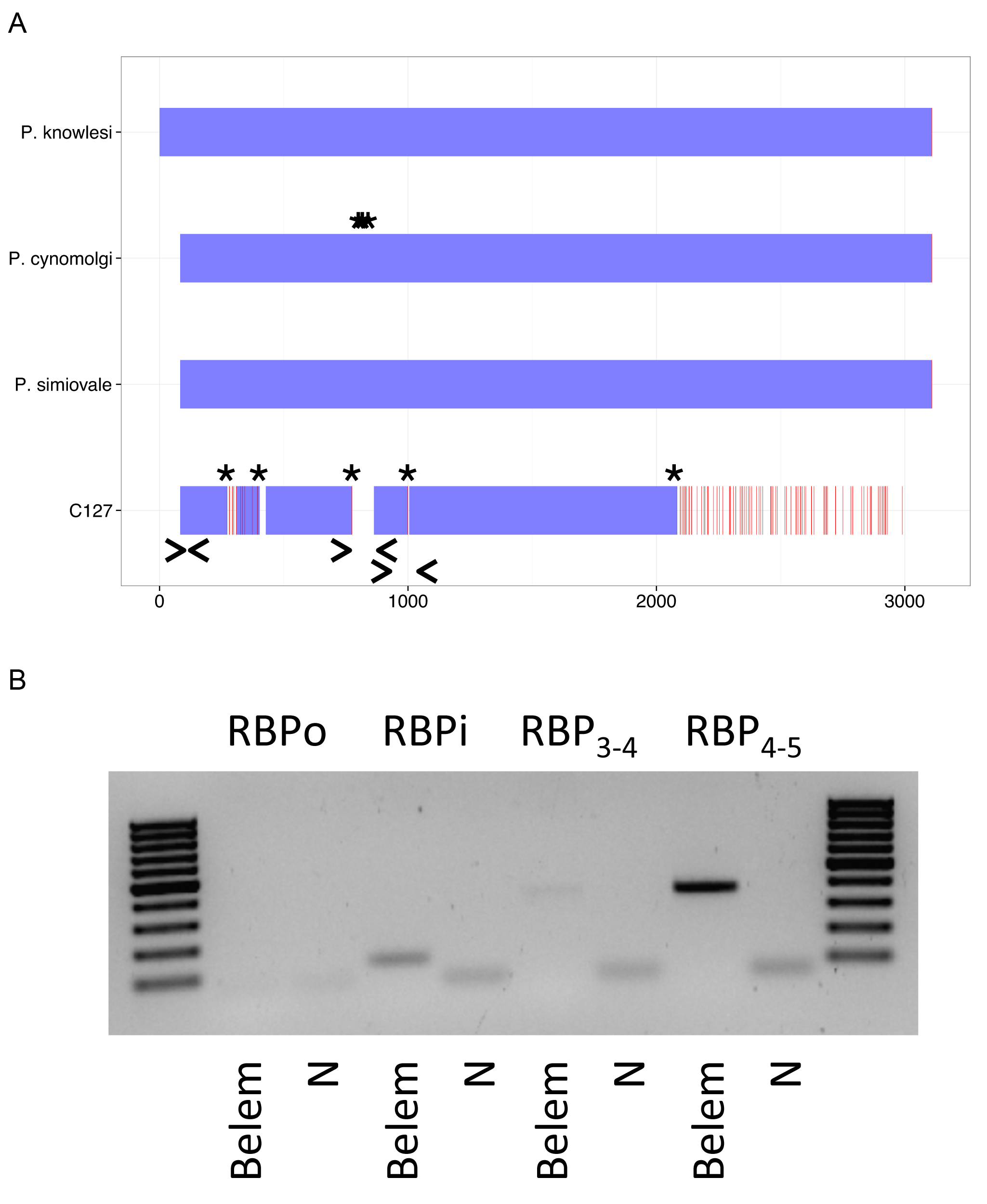

Supplement: Figure S6 — (A) Schematic representation of the predicted RBP2e proteins in different Plasmodium genomes. The solid blue boxes represent the predicted protein coding sequence in, from top to bottom, P. knowlesi, P. cynomolgi, P. simiovale and P. vivax (C127). The black asterisks indicate the locations of out-of-frame deletions that, in P. vivax, introduce stop codons (vertical red bars) and lead to multiple short predicted proteins. The black arrows indicate the primers used to amplify cDNA from the Belem strain (from left to right, RBPi, RBP3–4 and RBP4–5). (B) The gel picture shows the PCR products obtained for the P. vivax RBP2e gene using cDNA generated from the Belem strain. The leftmost amplification (RBPo) targets a region upstream of the predicted RBP2e transcript and fails to yield any PCR product. The next three amplifications all yield PCR products of the correct size and identical to the contig sequence after Sanger sequencing and correspond to i) amplification spanning an intron in the first predicted PvRBP2e gene (RBPi), ii) amplification between the 3rd and 4th predicted PvRBP2e genes (RBP3–4) and iii) amplification between the 4th and the 5th predicted PvRBP2e genes (RBP4–5). The primer sequences used are as follow: RBPoF 5′-CCTCTTCTAGCTATTGAACTCACCA-3′, RBPoR 5′-AGCGTGCATGGCTAATTGTA-3′, RBPiF 5′-TGTGATCTTTTGTAACCTCTTGTTT-3′, RBPiR 5′-TTCCCAAGGAAGTGGCATGT-3′, RBP3–4F 5′-TCCTGAGACGGTGGATAACA-3′, RBP3–4R 5′-TGTTCTTTAGCTGTGTGAGACT-3′, RBP4–5F 5-GGACTACGAGCAAAGTGCAG-3′, RBP4–5R 5′-AGCGGATTCTTTGTGACTCCTT-3′. (TIF) [file pntd.0002569.s006.tif]

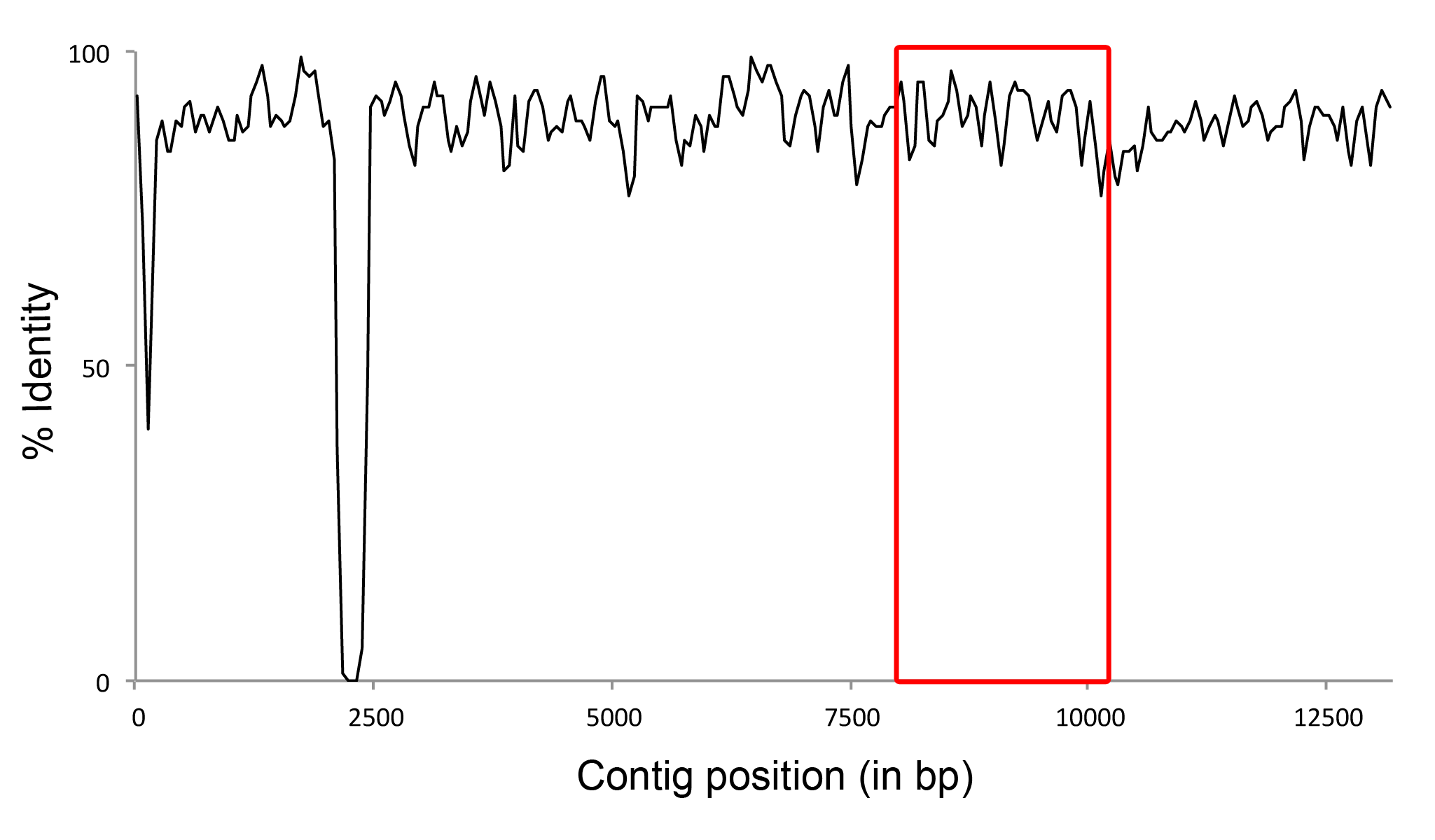

Supplement: Figure S7 — DNA sequence identity (y-axis, in %) between the C127 contig carrying the predicted EBP gene (indicated by the red box) and its orthologous P. cynomolgi contig (x-axis, in bp). (TIF) [file pntd.0002569.s007.tif]

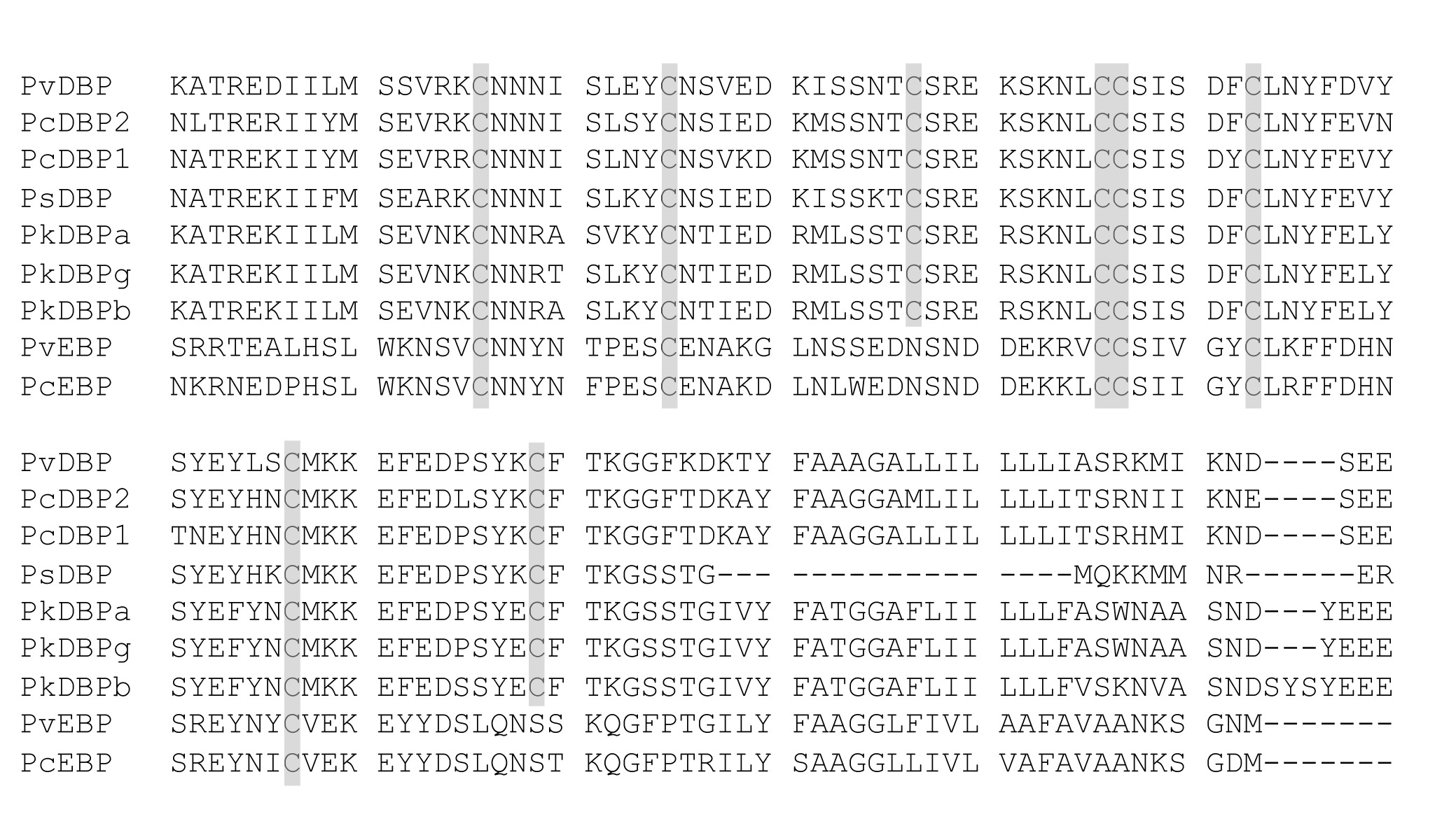

Supplement: Figure S8 — Amino acid alignment of the C-terminus cysteine-rich like domain for DBP genes and the novel P. vivax and P. cynomolgi EBP genes. The grey boxes indicate conserved cysteine positions. (TIF) [file pntd.0002569.s008.tif]

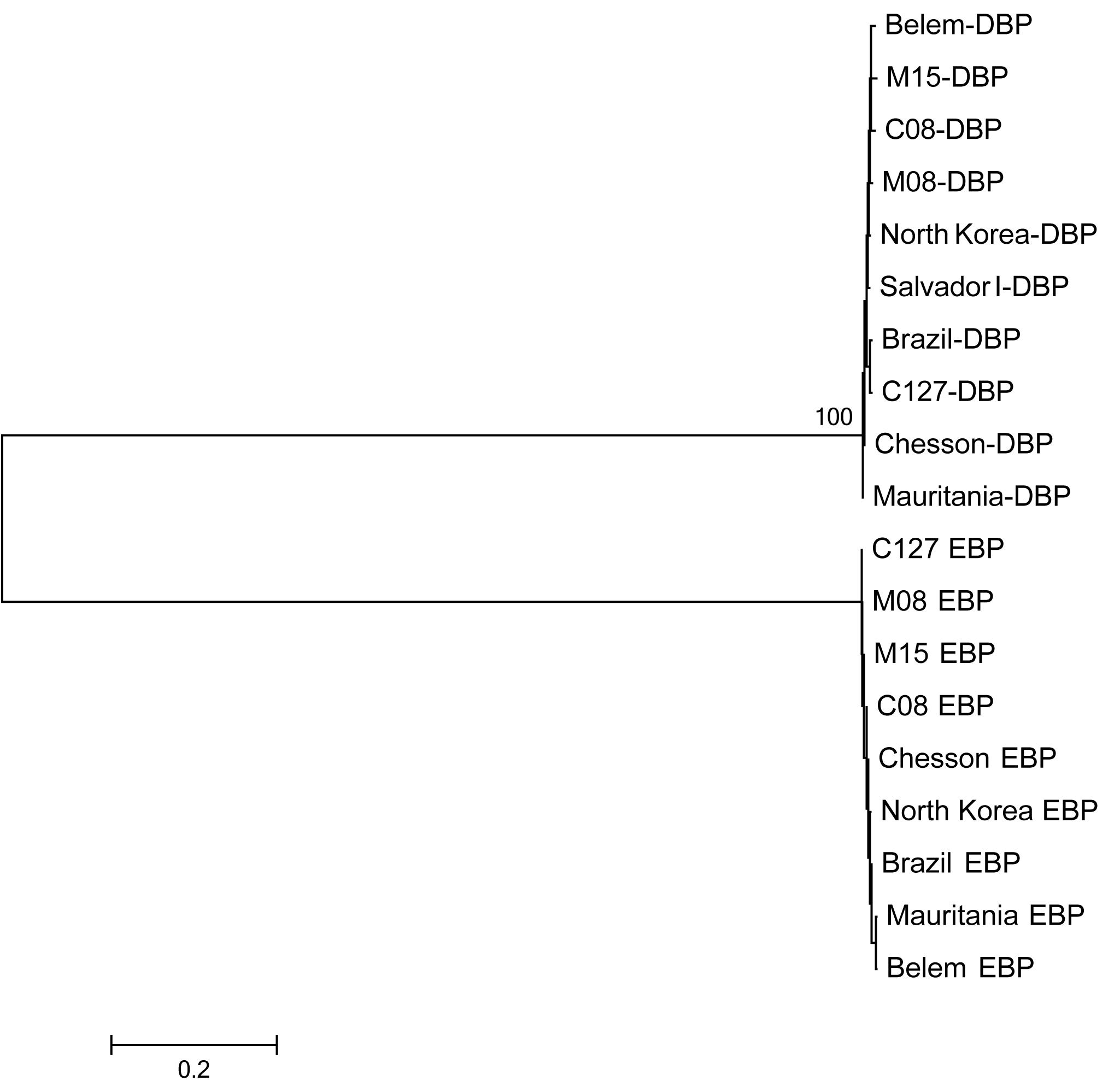

Supplement: Figure S9 — Phylogenetic tree showing the relationships between the amino-acid sequences of P. vivax samples for several RBP2 genes and the putative RBP2e. (TIF) [file pntd.0002569.s009.tif]
