# Supplementary material for: De Novo Assembly of a Field Isolate Genome Reveals Novel Plasmodium vivax Erythrocyte Invasion Genes
Source: PLoS Negl Trop Dis. 2013 Dec 5;7(12):e2569. doi: 10.1371/journal.pntd.0002569 (PMC3854868; doi:10.1371/journal.pntd.0002569)
Supplement: Table S2 — List of all contigs mapping partially to the Salvador I reference genome. (DOC) [file pntd.0002569.s011.doc]

| **Contig ID** | **Length (bp)** | **% Mapped to Salvador I** | **# of pred. genes** | **Mapped to** |
| --- | --- | --- | --- | --- |
| 30928 | 22,020 | 46 | 4 | Telomeric end of Chromosome 2 |
| 43113 | 46,268 | 88 | NA | Telomeric start of Chromosome 5 |
| 43364 | 59,163 | 83 | 7 | Center of Chromosome 5 |
| 46021 | 16,445 | 62 | 1 | Telomeric end of Chromosome 5 |
| 46685 | 36,000 | 52 | 4 | Telomeric start of Chromosome 7 |
| 48356 | 17,347 | 41 | 2 | Telomeric start of Chromosome 10 |
| 48376 | 27,804 | 47 | 2 | Telomeric end of Chromosome 12 |
| 48517 | 58,836 | 68 | 6 | Telomeric end of Chromosome 13 |
| 48684 | 78,610 | 77 | 4 | Telomeric start and end of Chr3 |
| 48904 | 12,706 | 50 | 1 | Chromosome 10 (MSP3 cluster) |
| 48915 | 120,006 | 96 | 1 | Telomeric start of Chromosome 13 |
| 49047 | 83,882 | 71 | 6 | Telomeric start of Chromosome 9 |
| 49146 | 54,278 | 87 | 1 | Telomeric start of Chromosome 4 |
| 49206 | 60,760 | 78 | 5 | Telomeric end of Chromosome 11 |
